# Targeted Mutagenesis of the Hypophysiotropic Gnrh3 in Zebrafish (*Danio rerio*) Reveals No Effects on Reproductive Performance

**DOI:** 10.1371/journal.pone.0158141

**Published:** 2016-06-29

**Authors:** Olivia Smith Spicer, Ten-Tsao Wong, Nilli Zmora, Yonathan Zohar

**Affiliations:** Department of Marine Biotechnology and Institute of Marine and Environmental Technology, University of Maryland, Baltimore County, Baltimore, Maryland, United States of America; Tel Aviv University, ISRAEL

## Abstract

Gnrh is the major neuropeptide regulator of vertebrate reproduction, triggering a cascade of events in the pituitary-gonadal axis that result in reproductive competence. Previous research in mice and humans has demonstrated that *Gnrh*/*GNRH* null mutations result in hypogonadotropic hypogonadism and infertility. The goal of this study was to eliminate *gnrh3* (the hypophysiotropic Gnrh form) function in zebrafish (*Danio rerio*) to determine how ontogeny and reproductive performance are affected, as well as factors downstream of Gnrh3 along the reproductive axis. Using the TALEN technology, we developed a *gnrh3*^*-/-*^ zebrafish line that harbors a 62 bp deletion in the *gnrh3* gene. Our *gnrh3*^*-/-*^ zebrafish line represents the first targeted and heritable mutation of a Gnrh isoform in any organism. Using immunohistochemistry, we verified that *gnrh3*^*-/-*^ fish do not possess Gnrh3 peptide in any regions of the brain. However, other than changes in mRNA levels of pituitary gonadotropin genes (*fshb*, *lhb*, and *cga*) during early development, which are corrected by adulthood, there were no changes in ontogeny and reproduction in *gnrh3*^*-/-*^ fish. The *gnrh3*^*-/-*^ zebrafish are fertile, displaying normal gametogenesis and reproductive performance in males and females. Together with our previous results that Gnrh3 cell ablation causes infertility, these results indicate that a compensatory mechanism is being activated, which is probably primed early on upon Gnrh3 neuron differentiation and possibly confined to Gnrh3 neurons. Potential compensation factors and sensitive windows of time for compensation during development and puberty should be explored.

## Introduction

In vertebrates, reproduction is regulated by the hypothalamus-pituitary-gonad (HPG) axis, which translates internal and external cues into endocrine signals and, ultimately, reproductive output. The axis’s control mechanisms include a complex network of neuropeptides that work at the level of the brain and/or the pituitary. Gonadotropin-releasing hormone (GNRH), the major regulator of neuroendocrine control of reproduction, stimulates the synthesis and release of the two pituitary gonadotropins, follicle-stimulating hormone (FSH) and luteinizing hormone (LH), that regulate steroidogenesis, gametogenesis, and final gamete maturation. During the past two decades, several neuropeptides that function upstream of GNRH and at the level of the pituitary, such as kisspeptin, neurokinin B, and gonadotropin-inhibitory hormone (GNIH), were discovered (for review, see [1] and also [2–4]).

GNRH was first discovered in the 1970s as an ovine and porcine neuropeptide capable of inducing the release of LH [5,6]. After demonstrating that this peptide also stimulates FSH release, the name was changed from luteinizing hormone-releasing hormone (LHRH) to GNRH. Usually, between one to three isoforms of GNRH can exist within a single species; however, apart from the species-specific hypophysiotropic form, the functions of the other two isoforms remain largely unknown. In most modern teleosts (e.g., perciforms), three isoforms of Gnrh exist: the species-specific hypophysiotropic Gnrh1 in the pre-optic area/hypothalamus, the ubiquitous (except for rodents) Gnrh2 in the midbrain tegmentum, and Gnrh3 in the terminal nerve/ventral telencephalon [7]. In some more primitive teleosts, such as salmonids and cyprinids (including the zebrafish, *Danio rerio*), only two Gnrh isoforms have been identified: Gnrh2 and Gnrh3 [8,9]. In these species, Gnrh3 is believed to assume the non-redundant roles of Gnrh1 [10] and has been demonstrated to be the hypophysiotropic form capable of inducing gonadotropin release [11,12].

The early development of zebrafish Gnrh3 begins with its expression in two cell clusters within the olfactory epithelium at approximately 24 hours post-fertilization (hpf) [11]. Shortly thereafter, fibers begin to extend from these cells that guide the Gnrh3 soma on a migration pattern that eventually leads to innervation of the pituitary at 4–5 days post-fertilization (dpf) [11,13]. In adult zebrafish, Gnrh3 soma are found along the terminal nerve/ventral telencephalon and in the pre-optic area/hypothalamus [11] with several projections throughout the brain and into the pituitary [13], where they interact with vasculature leading to the gonadotropes [14]. Earlier studies investigating the roles of Gnrh3 in the ontogeny of its neurons and in reproduction have established the importance of Gnrh3 in both processes. Gnrh3 knockdown by anti-sense morpholino oligonucleotides resulted in a misguided migration of Gnrh3 soma and fibers during early development, suggesting that Gnrh3 itself is needed for proper migration of its neurons [11]. In addition, successful laser ablation of Gnrh3-expressing cells in the olfactory region during early development (4–6 dpf) has led to the production of all females, in which oocyte development is arrested at stage II (cortical alveolus/pre-vitellogenic follicles), and infertility [12]. Therefore, it is believed that Gnrh3 neurons originating in the olfactory region during early development are essential to full gamete maturation and spawning, at least in females.

One of the most efficient approaches to better understand the roles that a particular gene plays on the survival and physiology of an organism is by loss-of-function studies, in which the functional product of the gene is eliminated. In 1977, Cattanach et al. [15] reported the discovery of a natural *Gnrh1* mutant mouse (*hpg*), in which individuals possess a 33.5 kb deletion that includes the latter half of the *Gnrh1* gene [16]. These *hpg* mice exhibit reduced pituitary content and circulating levels of FSH and LH and display hypogonadotropic hypogonadism, in which all individuals are sterile [15]. In addition, humans with hypogonadotropic hypogonadism are characterized by a failure to undergo puberty and have been described to possess one of approximately six different forms of mutations in *GNRH1* [17], in which one of the mutational “hot spots” tends to be in the region encoding the decapeptide [18]. Because efficient loss-of-function knockout techniques were not available in fish until recently, comparative experiments could not be conducted on teleosts to determine how the loss of Gnrh function manifests in physiological processes.

Recently, a new methodology for gene knockout has been introduced, which utilizes transcription activator-like effector (TALE) proteins combined to the non-specific nuclease FokI to produce TALE nucleases (TALENs). A TALEN can be designed to specifically induce a targeted double-stranded break in DNA. The result is a mutation in the gene of interest due to the error-prone nature of non-homologous end-joining [19]. The TALEN technology has been demonstrated to be far more successful than the previously used method for gene knockout in zebrafish (zinc-finger nucleases) due to the high degree of specificity of the TALE proteins. Thus far, TALENs have been used to successfully knockout genes in multiple animal species to elucidate gene functions [20–22], with much success in the zebrafish [23–25].

Because humans with *GNRH1* mutations [17], *hpg* mutant mice [15], and Gnrh3 cell-ablated zebrafish [12] are all infertile individuals with arrested gonad development, we hypothesized that knocking out the *gnrh3* gene in zebrafish would also lead to disrupted gametogenesis and the production of infertile fish. The goal of this study was to establish a *gnrh3*^*-/-*^ line of zebrafish to determine the mechanisms by which Gnrh3 exerts its regulatory functions. Specifically, we aimed at identifying the HPG components that are impacted by the loss of Gnrh3. We generated a *gnrh3*^*-/-*^ line, using the TALEN technology, and confirmed the lack of Gnrh3 using an antibody (generated for this purpose) against the zebrafish Gnrh3 Gnrh-associated peptide (Gap). The effects of *gnrh3* loss-of-function on reproductive factors downstream of Gnrh3 and reproductive competence were determined. Our results clearly show that, unlike in mice and humans, the absence of Gnrh3 had very few effects on the downstream components of the HPG axis. Detailed comparisons between the *gnrh3*^*+/+*^ and *gnrh3*^*-/-*^ lines are provided, and possible explanations for the lack of the expected phenotypes are discussed.

## Materials and Methods

### Animals

All zebrafish used in this study originated from the in-house colony at the Institute of Marine and Environmental Technology in Baltimore, MD. Zebrafish were maintained in a recirculating system at 28°C with a photoperiod of 14-h light and 10-h dark and were monitored and fed twice daily with a commercial flake food or pellets *ad libitum*. Zebrafish embryos and larvae were raised in individual containers of freshwater until 30 dpf, before being transferred to the recirculating system. Starting at 5 dpf, larval zebrafish were fed *Paramecium* twice daily, until 14 dpf, when *Artemia* nauplii was introduced to their diet. Prior to tissue collections, adult fish were euthanized by immersion in an ice bath followed by quick decapitation. All experimental protocols were approved by the Institutional Animal Care and Use Committee at the University of Maryland Baltimore School Of Medicine.

### Preparation and Microinjection of TALEN mRNAs

A pair of zebrafish *gnrh3*-targeting TALENs were designed to induce a double-stranded gDNA break at base pair 3700 in *gnrh3* gDNA (Ensembl ENSDARG00000056214) or base pair 71 of the *gnrh3* coding region (NCBI Reference Sequence NM_182887.2) by an NIH-sponsored initiative (NIH R01 GM088040). This TALEN target site is the second base pair of the codon that encodes for the first amino acid (glutamine) in the zebrafish Gnrh3 decapeptide, which is located within the recognition site of the Cac8i restriction enzyme, allowing a useful way to screen for mutated gDNA. Each *gnrh3*-targeting TALEN sequence was cloned into the JDS71 vector under the control of the T7 promoter (Addgene). The TALEN plasmids were linearized with PmeI, and one μg of each linearized plasmid was transcribed into mRNA with the Ambion mMessage mMachine® Transcription Kit, using the T7 RNA polymerase. After DNase treatment, a polyA tail was added to the mRNA samples (Ambion PolyA Tailing Kit).

Approximately 50–75 ng/μL of both TALEN mRNAs (left and right) were simultaneously micro-injected into 1- to 2-cell stage WT zebrafish embryos (F0), which were grown to maturity. To identify fish that carry a mutation, sperm (~0.5–1 μL) was collected by stripping each male individual, resuspended in 10 μL HBSS, and used as a PCR screening template using TALEN-targeted primers. PCR products were digested with Cac8I and run on a 2% agarose gel to distinguish between mutant and WT DNA. Genomic DNA PCR products that were not digested by Cac8I were assumed to be mutated. The undigested (mutant DNA) products were gel-extracted, purified, and sequenced.

### Development of a *gnrh3*^*-/-*^ Line

F0 males that contained a *gnrh3*-specific mutation were crossed with WT females to obtain F1 offspring. Once mature, F1 fish were fin-clipped for gDNA extraction. Briefly, caudal fin tissue was immersed in gDNA extraction buffer (50 mM KCl, 10 mM Tris-HCl (pH = 8.0), 150 mM MgCl_2_, 0.3% Tween-20, and 0.3% NP40 in sterile MilliQ water), boiled at 95–100°C for 15 minutes, cooled on ice, and digested with proteinase K (New England BioLabs) at 55°C for 1–3 hours. After digestion, lysates were boiled at 95–100°C for 15 minutes to inactivate proteinase K and centrifuged for 3 minutes at 12,000 rpm. The resulting gDNA was genotyped by PCR and Cac8I digestion, as described above.

F1 adults that contained a *gnrh3* mutation were crossed with WT fish to obtain heterozygous fish (F2). Heterozygous adults were in-crossed to obtain the F3 generation, of which ¼ were WT (*gnrh3*^*+/+*^), ½ were heterozygous for the *gnrh3* mutation (*gnrh3*^*+/-*^), and ¼ were homozygous for the *gnrh3* mutation (*gnrh3*^*-/-*^). All F3 *gnrh3*^*-/-*^ fish were verified by a two-step PCR and sequencing of fin-clip gDNA. The first step involves a double PCR reaction, in which two different forward primers (Step1mutantFor and Step1WTFor), along with the same reverse primer (StepRev), compete for the mutated or WT gDNA, respectively. The results from step 1 distinguish between *gnrh3*^*+/+*^, *gnrh3*^*+/-*^, and *gnrh3*^*-/-*^ gDNA. The second step PCR (primers Step2For and StepRev) is conducted on the *gnrh3*^*-/-*^ gDNA and amplifies a larger product for sequencing. The primers for the two-step PCR can be found in Table 1.

**Table 1:** PCR primers for a two-step PCR process used to screen F3 fish fin-clip gDNA as either *gnrh3*^*+/+*^, *gnrh3*^*+/-*^, or *gnrh3*^*-/-*^ and to submit for sequencing.

Primer	PCR Step	Type	Sequence (5' → 3')	T_m_ (°C)	GC %	Amplicon size (bp)*
Step1mutantFor	1	Mutant For	TTAGTTTAGATTTCAGCAGTTTTAGCATCATA	55.3	28.1	544
Step1WTFor	1	WT For	GAAGGTTGCTGGTCCAGTTGTTGCTG	62.2	53.8	582
Step2For	2	For	TTGAGATGGAAGACAATCCTTT	51.9	36.4	800
StepRev	1 and 2	Rev	TGCACATGTACTTGCTGAATTA	52.6	36.4	

*when paired with StepRev primer.

### Generation of Antibody against Zebrafish Gnrh3 Gnrh-Associated Peptide (Gap)

The cDNA encoding zebrafish Gnrh3 Gap (from 136 bp to 309 bp of NCBI Reference Sequence NM_182887.2) was cloned into the pET-15b vector and expressed in Rosetta-gami B(DE3)pLysS *E*. *coli* cells (Novagen) as N-terminal His-tagged recombinant proteins. The proteins from the insoluble fraction were prepared according to Brent [26]. These proteins were dissolved in 8M urea, purified with nickel-nitrolotriacetic acid columns (Promega), and de-salted on sephadex G-15 columns (Pharmacia). The purified proteins were used for the production of antiserum in rabbits (ProteinTech). The final bleed antiserum was used as the primary polyclonal antibody in all zebrafish Gnrh3 Gap immunohistochemistry.

### Verification of Anti-zebrafish Gnrh3 Gap Antibody

The specificity of the zebrafish Gnrh3 Gap antibody was verified by confirming the recognition and specificity of the antibody to Gnrh3 expressed in a heterologous cell line and by co-localizing Gnrh3 immunoreactivity in *gnrh3* transgenic adult brain sections (as described in next section), expressing tdTomato in *gnrh3* neurons [13]. The entire zebrafish *gnrh3* coding region (from 28 bp to 312 bp of NCBI Reference Sequence NM_182887.2) was cloned into the pcDNA3.1 (Life Technologies) mammalian expression vector under the control of the CMV promoter. The zebrafish *gnrh3*-pcDNA3.1 plasmid and the control pcDNA3.1 plasmid were transfected into COS7 cells with FuGENE 6.0 (Promega). As an additional control to check for cross-reactivity, we also transfected cells with the zebrafish *gnrh2*-pcDNA3.1 plasmid. The cells were grown in 25 cm^2^ sterile cell culture flasks in DMEM supplemented with 10% FBS, 1% glutamine, 100 U/mL penicillin, and 100 mg/mL streptomycin (Biological Industries) at 37°C and 5% CO_2_. After 48 hours of incubation, cells were transferred to chamber slides. After 24 hours, the cells were fixed with 4% paraformaldehyde (PFA) in PBS for 1 hour at room temperature and were washed with PBS. Blocking and immunostaining for zebrafish Gnrh3 Gap was then conducted as described in the next section.

### Gnrh3 Immunohistochemistry

Brains were dissected from adult fish and fixed overnight with 4% PFA (in PBS) at 4°C. Before cryopreservation, tissues were transferred to 30% sucrose (in PB) overnight at 4°C. Brain tissues were frozen in OCT, coronally sectioned at 15 μm thickness, transferred to charged slides, and stored at -20°C until immunohistochemistry. Briefly, dried sections were fixed in pre-chilled acetone for 2 minutes. Sections were blocked with 5% goat serum and 0.3% Triton X-100 in PBS for 1 hour at room temperature. Sections were incubated overnight at 4°C with rabbit anti-zebrafish Gnrh3 Gap (1:1000–1:3000) diluted in 1% BSA and 0.3% Triton X-100 in PBS. After PBS washes with 0.3% Triton X-100, sections were incubated for 1 hour at room temperature with goat anti-rabbit secondary antibody Alexa 488 (Life Technologies) or Cy3 (KPL, Inc.) diluted 1:300–1:500 in 1% BSA and 0.3% Triton X-100 in PBS.

To provide more verification of the lack of Gnrh3 protein in the *gnrh3*^*-/-*^ fish, we also immunostained *gnrh3*^*+/+*^ and *gnrh3*^*-/-*^ adult brain sections with an additional polyclonal primary antibody (anti-Gnrh3 decapeptide, kindly provided by Dr. Judy King). Before blocking and overnight incubation with the primary antibody (diluted 1:700), slides were quenched with 0.3% H_2_O_2_ in PBS for 30 minutes. Visualization of the protein signal required the Tyramide Signal Amplification (TSA; Perkin Elmer) Plus kit, according to the manufacturer’s protocol. Goat anti-rabbit-HRP (diluted 1:1000) was used to detect the primary antibody, and fluorescence was obtained via Cy3 (red) from the kit. Slides were mounted with Vectashield with DAPI (Vector Labs) and imaged with a Zeiss Axioplan 2 microscope with an Attoarc HBO100 W power source, equipped with a CCD Olympus DP70 camera. All images were taken at 20x magnification with a resolution of 1360 x 1024 and analyzed with Adobe Photoshop.

### *gnrh3* in situ Hybridization

Brains were dissected from adult *gnrh3*^*+/+*^ and *gnrh3*^*-/-*^ fish and fixed overnight with 4% PFA (in PBS) at 4°C. Before cryopreservation, tissues were transferred to 30% sucrose (in PB) overnight at 4°C. Brain tissues were frozen in OCT, sagitally sectioned at 10 μm thickness, transferred to charged slides, and stored at -80°C until in situ hybridization. Anti-sense and sense DIG-labeled riboprobes were synthesized from the cDNA clone of the *gnrh3* coding region (from 28 bp to 312 bp of NCBI Reference Sequence NM_182887.2), using RNA polymerase (Roche Diagnostics). The in situ hybridization protocol was followed as according to Zmora et al. [27] with a riboprobe concentration of 500 ng/mL. The signal was detected using the TSA Plus kit, according to the manufacturer’s protocol and using anti-DIG HRP (diluted 1:200; Roche Diagnostics). Fluorescence was obtained via the Cy3 dye from the TSA kit. Negative control slides were similarly treated with sense riboprobes. Slides were mounted with anti-fading solution with DAPI and imaged with a Zeiss Axioplan 2 microscope with an Attoarc HBO100 W power source, equipped with a CCD Olympus DP70 camera. All images were taken at 20x magnification with a resolution of 1360 x 1024 and analyzed with Adobe Photoshop.

### Developmental and Adult Gene Expression Profiles of *gnrh3*^*-/-*^ Fish

In order to characterize the *gnrh3*^*-/-*^ line during development, *gnrh3*^*+/+*^ and *gnrh3*^*-/-*^ embryos/larvae/juveniles were sampled at 8 developmental time points (1, 2, 3, 8, 12, 18, 24, and 30 dpf). All samplings were done in triplicate. Ten *gnrh3*^*+/+*^ and ten *gnrh3*^*-/-*^ embryos or larvae each were pooled and collected at each sampling point from 1 dpf to 18 dpf, whereas 8 larvae and 6 larvae were pooled and collected at 24 dpf and 30 dpf, respectively. Embryos/larvae/ juveniles were frozen on dry ice and stored at -80°C until RNA extraction. During adulthood, four *gnrh3*^*+/+*^ and four *gnrh3*^*-/-*^ fish of reproductive age were selected from each sex for dissection of the brains, pituitaries, and gonads. Tissues were frozen on dry ice and stored at -80°C until RNA extraction.

Total RNA was extracted from tissues using TRIzol® reagent (Invitrogen), according to the manufacturer’s protocol, and total RNA was quantified with a Nanodrop (Thermo Scientific). One μg total RNA was treated with gDNA wipeout buffer for 9 min at 42°C and synthesized into first-strand cDNA with the Qiagen QuantiTect Reverse Transcriptase (RT) Kit in a 20 μL reaction. QPCR was conducted for the following genes: brain: *gnrh2*, *gnrhr1*, *gnrhr2*, *gnrhr3*, *gnrhr4*, *cyp19a2*; pituitary: *fshb*, *lhb*, *cga*, *gnrhr1*, *gnrhr2*, *gnrhr3*, *gnrhr4*; and gonad: *fshr* and *lhr*. Gene-specific QPCR primers (for genes in which major differences were observed) are listed in Table 2, with *ef1a* as an internal control. The efficiency of each QPCR primer set, as determined by the standard curve R^2^, was R^2^ ≥ 0.951 for the developmental time series and R^2^ ≥ 0.988 for adult pituitaries. Each QPCR reaction was carried out in duplicate with a final volume of 10 μL: 2x DyNAmo Flash SYBR Green QPCR mix (Life Technologies), 200 nM primer mix, 0.3x ROX (Life Technologies), 20 ng cDNA, and sterile MilliQ water, in a 7500 Fast Real-Time PCR System (Applied Biosystems, Inc.). The cycle conditions were 95°C for 7 minutes, followed by 40 cycles of 95°C for 10 seconds and 60°C for 30 seconds. Each plate included a standard curve using transcribed RNA of the specific clone as a template, and absolute copy number was calculated for the tested reactions and normalized against the house-keeping gene *ef1a*. Each plate included two no RT controls and two no template controls.

**Table 2:** QPCR primers used to quantify gene transcripts in *gnrh3*^*+/+*^ and *gnrh3*^*-/-*^ fish.

Gene	Type	Sequence (5’ → 3’)	T_m_ (°C)	GC%	Amplicon Size (bp)
*fshb*	For	GCTGGACAATGGATCGAGTTTA	54.9	45.5	92
*fshb*	Rev	CTCGTAGCTCTTGTACATCAAGTT	54.5	41.7	
*lhb*	For	GGCTGGAAATGGTGTCTTCT	55.1	50.0	107
*lhb*	Rev	CCACCGATACCGTCTCATTTAC	55.1	50.0	
*cga*	For	TCCGGTCTATCAGTGCGT	55.6	55.6	148
*cga*	Rev	GGATATTCGTGGCAACCATTT	53.5	42.9	
*ef1a*	For	AAGACAACCCCAAGGCTCTCA	58.6	52.4	255
*ef1a*	Rev	CCTTTGGAACGGTGTGATTGA	55.5	47.6	

### Lh ELISA

Four pituitaries from adult *gnrh3*^*+/+*^ and *gnrh3*^*-/-*^ males and females were analyzed by ELISA for Lh content. The protocol was originally developed for carp Lh [28], based on the procedure described for tilapia (*Oreochromis niloticus*) Lh [29], and adopted for zebrafish Lh [30]. For the carp ELISA, which was used for the current study, the intra-assay and inter-assay coefficients of variation were 7.6% and 11.3%, respectively, while the sensitivity of the assay was 32 pg/mL [28].

### Imaging of Gnrh3 Fibers in *gnrh3*^*-/-*^ *gnrh3*:*tdTomato* Juveniles

We crossed our *gnrh3*^*+/+*^ and *gnrh3*^*-/-*^ fish with the *gnrh3*:*tdTomato* transgenic line previously developed in our lab [11,13]. Fish that were heterozygous for both the *gnrh3* mutation and the *gnrh3*:*tdTomato* transgene were in-crossed to obtain fish that were homozygous for both the mutation and the transgene. Importantly, *gnrh3*^*-/-*^ fish that express *gnrh3*:*tdTomato* do not express the Gnrh3 decapeptide, since the *gnrh3*:*tdTomato* transgene does not include the portion of exon 2 in *gnrh3* that encodes for the Gnrh3 decapeptide.

At 34 dpf, *gnrh3*^*+/+*^ *gnrh3*:*tdTomato* and *gnrh3*^*-/-*^ *gnrh3*:*tdTomato* juveniles were fixed overnight in 4% PFA (in PBS) at 4°C and then decalcified for 5–7 days in 0.5M EDTA (pH = 8.0) at 4°C. Before cryopreservation, tissues were transferred to 30% sucrose (in PB) overnight at 4°C. Fish were frozen in OCT, sagitally sectioned at 30 μm thickness, transferred to charged slides, and stored at -20°C. After slides were dried, washed with PBS, and mounted with anti-fading solution with DAPI, slides were viewed and imaged with a Leica Microsystems DMi8 confocal microscope. Z-stack images were taken at 20x magnification and at a resolution of 1024 x 1024 with a z-step size of 0.10. All images were analyzed and assembled with Image J and Adobe Photoshop.

### Gonad Histology

Whole gonads were fixed overnight in 4% PFA (in PBS) at 4°C, dehydrated with an ethanol series, cleared with two xylene washes, embedded in paraffin, sectioned at 5 μm on a HM 340 microtome (Thermo Scientific), and allowed to dry overnight at 42°C. Sections were stored at 4°C until paraffin removal and tissue hydration with two xylene washes and an ethanol series. Sections were stained with hematoxylin and eosin and mounted with Permount. Slides were analyzed and imaged with the brightfield setting of a Zeiss Axioplan 2 microscope, equipped with a CCD Olympus DP70 camera, to determine the most advanced stages of oogenesis or spermatogenesis. All images were taken at 20x or 5x magnification for males and females, respectively, with a resolution of 1360 x 1024.

### Fecundity/Fertility/Offspring Survival Assessments

In order to determine fecundity, fertility, and offspring survival of *gnrh3*^*-/-*^ fish, three to six spawning pairs of each of the following were used to obtain offspring: a) *gnrh3*^*+/+*^ male x *gnrh3*^*+/+*^ female, b) *gnrh3*^*+/+*^ male x *gnrh3*^*-/-*^ female, c) *gnrh3*^*-/-*^ male x *gnrh3*^*+/+*^ female, and d) *gnrh3*^*-/-*^ male x *gnrh3*^*-/-*^ female. A male and female fish were placed in a spawning container the night before spawning and were kept separated until the divider was removed immediately after lights on in the morning. One hour after removing the divider, embryos were collected and counted for each pair to determine fecundity. At 4–5 hpf, viable embryos were counted for each pair to determine the percentage of fertilization. Embryos were then assessed for percentage of survival at 2 dpf.

### Statistics

All data is represented as mean values ± standard error of the mean (SEM). For QPCR and ELISA assays, statistical analyses were conducted by using a one-tailed Student *t*-test, assuming equal variance. In addition, for QPCR of the developmental profiles, a two-way ANOVA was also conducted for both sample time and genotype with Minitab 17. For analysis of fecundity, fertility, and offspring survival data, a one-way ANOVA was conducted with GraphPad Instat 3. Statistical significance was established if *P values ≤ 0.05.

## Results

### Generation and Characterization of the *gnrh3*^*-/-*^ Line

#### Development of *gnrh3-/-* Line

**Fig 1:**
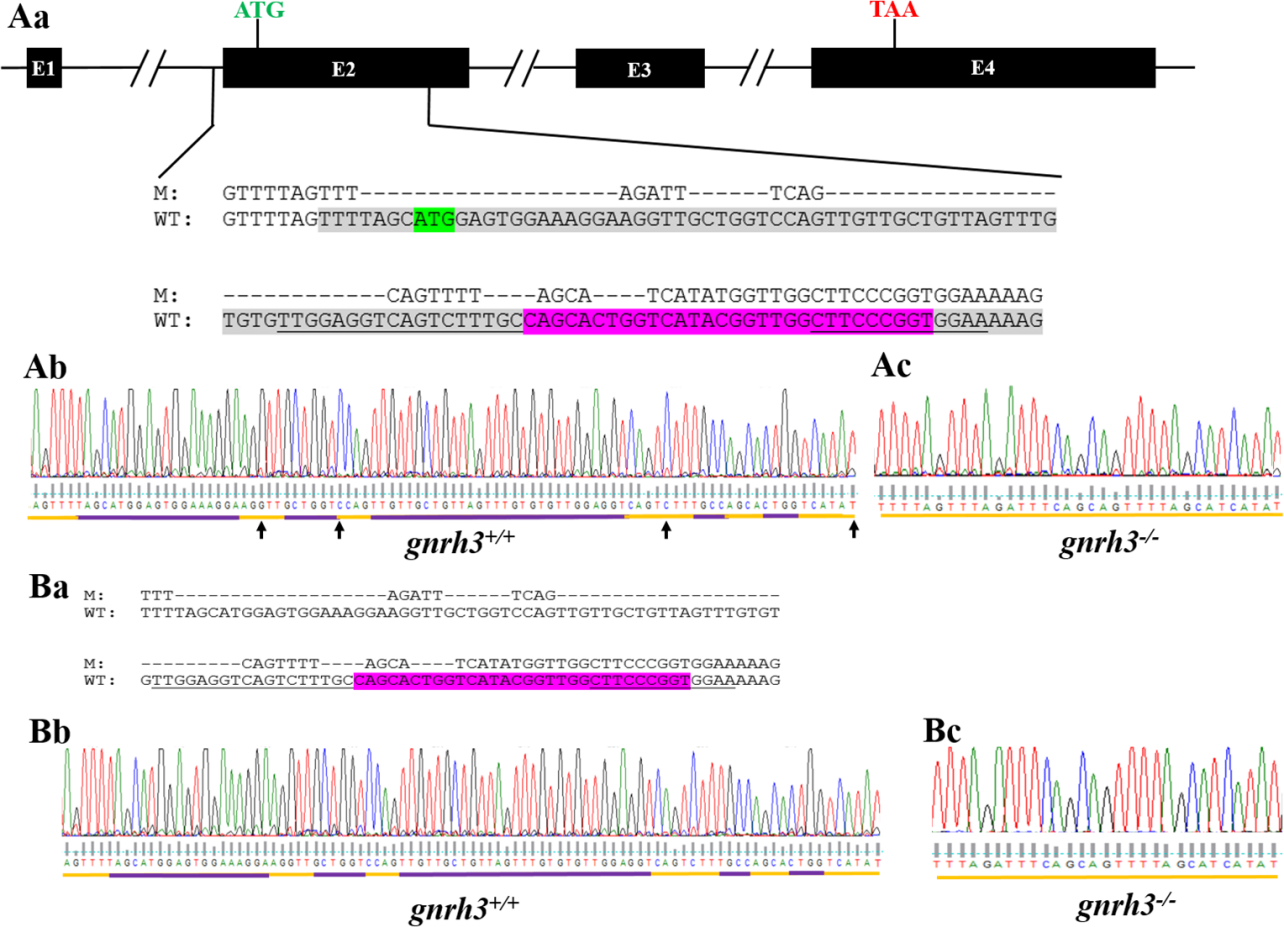
Verification of targeted, heritable mutation in *gnrh3* by gene and transcript. (Aa) The *gnrh3*^*-/-*^ deletion is localized to exon 2 (highlighted in gray) of the *gnrh3* gene and includes the start codon (highlighted in green) and a portion of the nucleotide sequence encoding the Gnrh3 decapeptide (highlighted in pink). The underlined regions represent the TALEN recognition sites. WT = *gnrh3*^*+/+*^. M = *gnrh3*^*-/-*^. (Ab-c) Sequencing chromatograms of *gnrh3*^*+/+*^ and *gnrh3*^*-/-*^ gDNA, demonstrating the homogenous sequence of the *gnrh3*^*-/-*^ mutation. The purple regions represent the gDNA sequences of *gnrh3*^*+/+*^ that were deleted in the *gnrh3*^*-/-*^ gene. Black arrows represent where single base pair substitutions occurred. (B) The *gnrh3*^*-/-*^ cDNA reveals no change in the *gnrh3*^*-/-*^ mutation after transcription. (Ba) The pink region represents the nucleotide sequence of the Gnrh3 decapeptide, while the underlined regions represent the TALEN recognition sites. WT = *gnrh3*^*+/+*^. M = *gnrh3*^*-/-*^. (Bb-c) Chromatograms of *gnrh3*^*+/+*^ and *gnrh3*^*-/-*^ cDNA indicate the homogenous sequence of the *gnrh3*^*-/-*^ mutation.

Following PCR screening for the mutated *gnrh3* gene in the F1 generation, two mutated sequences were detected: a 9 bp continuous deletion within the Gnrh3 decapeptide nucleotide sequence (data not shown) and a 62 bp discontinuous deletion, which included the ATG start codon and the beginning of the nucleotide sequence encoding the Gnrh3 decapeptide (Fig 1Aa). The 62 bp deletion was restricted to exon 2 and included four single bp substitutions (black arrows in Fig 1Ab). F1 adults with the 62 bp deletion were crossed to WT fish to obtain heterozygous F2 fish (*gnrh3*^*+/-*^). Heterozygotes were in-crossed, and one-quarter of their offspring were determined to be *gnrh3*^*-/-*^ by PCR and sequencing (Fig 1Ac). The results also revealed the same 62 bp deletion in the *gnrh3*^*-/-*^ cDNA (Fig 1Ba), which was also determined to be homogeneous (Fig 1Bc). Because the mutation in the gDNA is completely located within exon 2, the sequence of the cDNA mutation was identical to that of the gDNA (Fig 1Aa vs Fig 1Ba). These results demonstrated no alternative splicing in the *gnrh3*^*-/-*^ mRNA.

#### Verification of *gnrh3-/-* Line

**Fig 2:**
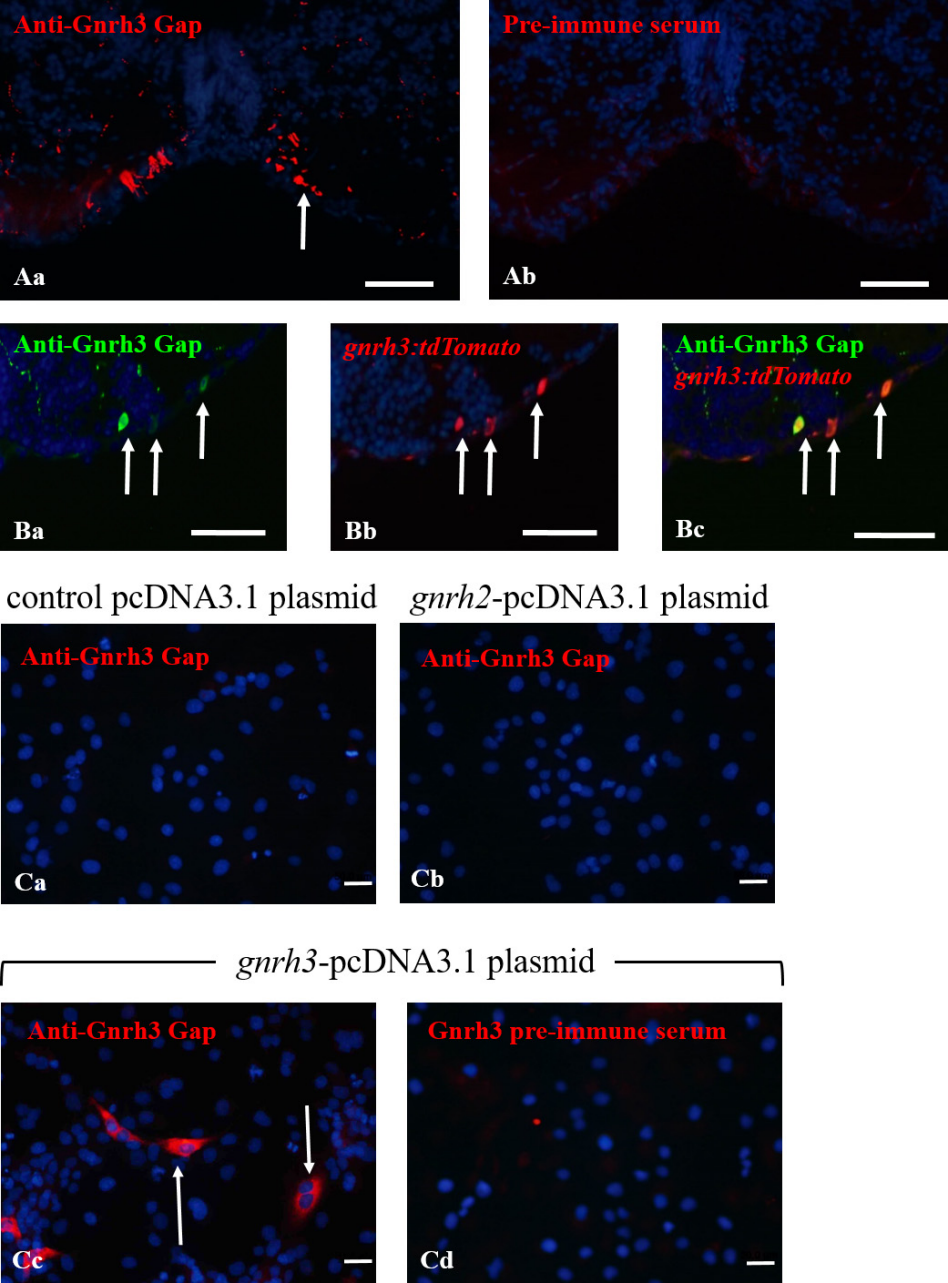
Anti-zebrafish Gnrh3 Gap polyclonal antibody is specific to zebrafish Gnrh3. (A) The immunostaining of Gnrh3-ir soma and fibers (white arrow; Aa) in the brain with anti-zebrafish Gnrh3 Gap was eliminated when the antibody was substituted with pre-immune serum (Ab). (B) Immunostaining with anti-Gnrh3 Gap (green; Ba) on brain sections of *gnrh3*:*tdTomato* (red; Bb) adults shows co-localization between Gnrh3-ir soma and *gnrh3*:*tdTomato*-labeled soma (Bc), indicating the specificity of the antibody to Gnrh3. White arrows indicate Gnrh3-tdTomato-expressing soma that were positively stained by anti-Gnrh3 Gap. (C) Immunostaining with (Ca-Cc) anti-zebrafish Gnrh3 Gap or (Cd) pre-immune serum in COS7 cells transfected with (Ca) control pcDNA3.1 plasmid, (Cb) *gnrh2*-pcDNA3.1 plasmid, or (Cc,d) *gnrh3*-pcDNA3.1 plasmid. The cells that express zebrafish Gnrh3 and are immunostained with anti-zebrafish Gnrh3 Gap (red) are indicated by white arrows (Cc). Scale bars = 50 μm.

In order to demonstrate that the *gnrh3*^*-/-*^ line indeed lacks Gnrh3 expression at the protein level, the anti-zebrafish Gnrh3 Gap polyclonal antibody was developed. The anti-zebrafish Gnrh3 Gap antibody stained soma and fibers in the *gnrh3*^*+/+*^ forebrain (Fig 2Aa), while the negative control pre-immune serum did not yield any positive signal in the same region (Fig 2Ab). We also conducted Gnrh3 immunohistochemistry on brain sections of adult *gnrh3*:*tdTomato* fish to look for co-localization between the two signals. In the forebrain, positive Gnrh3 immunostaining of soma (Fig 2Ba) was observed coinciding with Gnrh3 neurons expressing Gnrh3-tdTomato (Fig 2Bb), indicating that the cells in which activation of the *gnrh3* promoter occurs are also recognized by the anti-Gnrh3 Gap antibody (Fig 2Bc). The antibody also specifically recognized Gnrh3 peptide expressed in COS7 cells (Fig 2Cc) in vitro, while Gnrh2-expressing cells (Fig 2Cb) or cells carrying the control pcDNA3.1 plasmid (Fig 2Ca) were not stained. Therefore, our polyclonal antibody specifically recognizes the zebrafish Gnrh3 Gap and does not cross-react with the Gnrh2 Gap.

**Fig 3:**
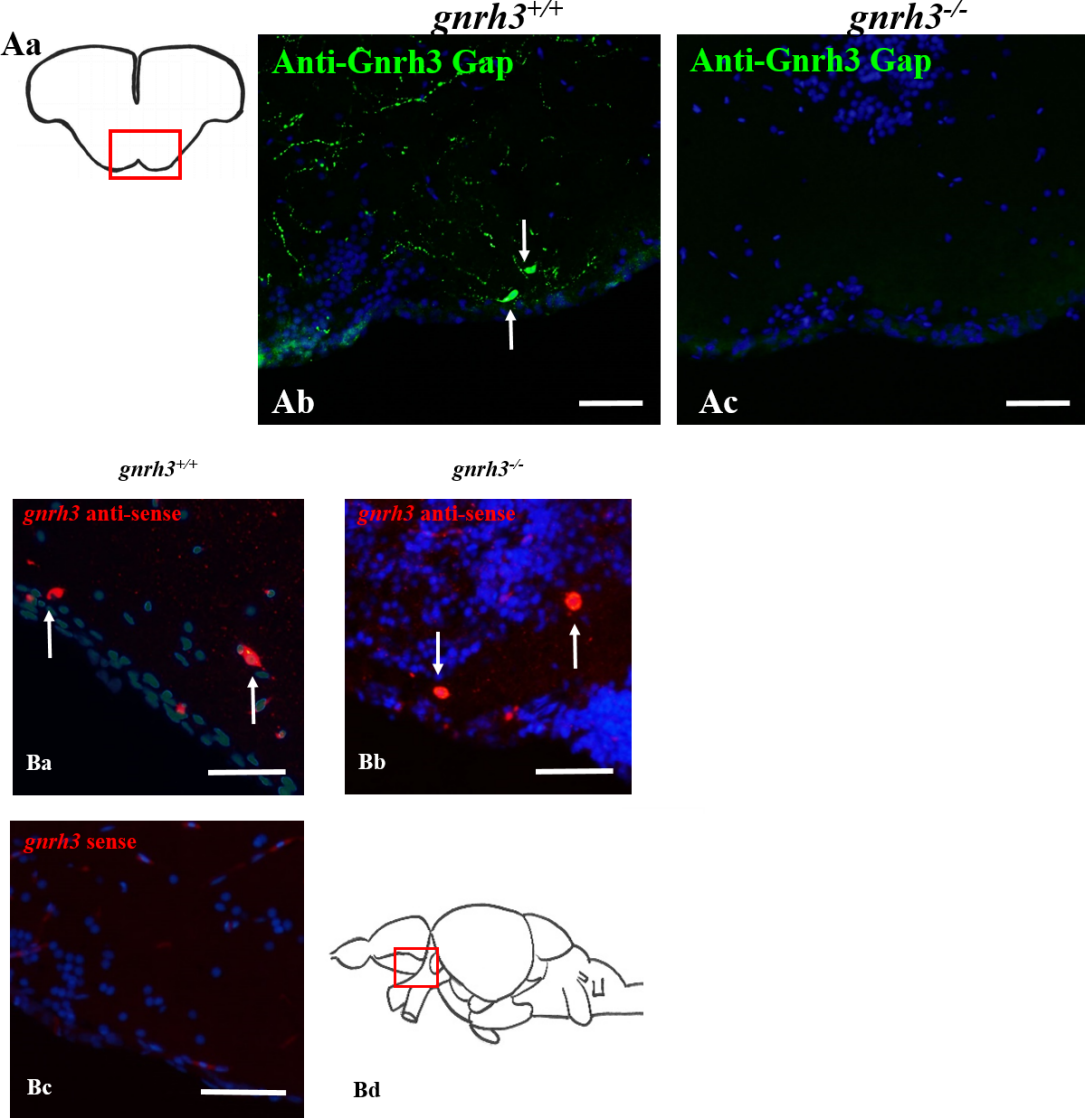
*gnrh3* mRNA but not Gnrh3 protein detectable in *gnrh3*^*-/-*^ fish. **(**A) Immunohistochemistry on adult coronal brain sections (Aa) using anti-zebrafish Gnrh3 Gap (green) demonstrates the presence of Gnrh3 signal in the form of somas (white arrows) and fibers in the *gnrh3*^*+/+*^ pre-optic area of the brain (Ab). However, no Gnrh3 signal was found in the *gnrh3*^*-/-*^ pre-optic area (Ac) or in any other region of the brain. (B) In situ hybridization on adult sagittal brain sections (Bd) using *gnrh3* DIG-labeled riboprobes. The anti-sense *gnrh3* riboprobe demonstrated mRNA (red) in the ventral telencephalon and pre-optic area of both *gnrh3*^*+/+*^ (Ba) and *gnrh3*^*-/-*^ (Bb) fish. The sense *gnrh3* riboprobe demonstrated no *gnrh3* mRNA signal in any brain regions in the *gnrh3*^*+/+*^ fish (Bc). Scale bars = 50 μm.

After obtaining a specific anti-zebrafish Gnrh3 Gap polyclonal antibody, immunohistochemistry was conducted on adult *gnrh3*^*+/+*^ and *gnrh3*^*-/-*^ brain sections (Fig 3A). Immunostaining with this primary antibody revealed positive staining of soma and fibers within the pre-optic area of coronal brain sections of *gnrh3*^*+/+*^ fish (Fig 3Ab). However, immunostaining of *gnrh3*^*-/-*^ brain sections did not reveal any signal in the pre-optic area (Fig 3Ac) or in any other regions of the brain. Immunostaining with a polyclonal anti-Gnrh3 decapeptide antibody developed in rabbit (kindly provided by Dr. Judy King) also revealed a lack of signal in the *gnrh3*^*-/-*^ adult brains (S1 Fig). Thus, the established *gnrh3*^*-/-*^ line indeed lacks any Gnrh3, rendering it a complete knockout line of *gnrh3*.

Although the *gnrh3*^*-/-*^ fish demonstrate a lack of Gnrh3, *gnrh3* in situ hybridization was conducted to determine if *gnrh3* mRNA is still produced in *gnrh3*^*-/-*^ fish and recognized by a *gnrh3* anti-sense riboprobe. Both *gnrh3*^*+/+*^ and *gnrh3*^*-/-*^ brain sections treated with the *gnrh3* anti-sense riboprobe yielded positive signal in soma located in the ventral telencephalon and preoptic area (Fig 3Ba and 3Bb). Sections of *gnrh3*^*+/+*^ fish treated with the *gnrh3* sense riboprobe did not yield any signal (Fig 3Bc). Therefore, *gnrh3*^*-/-*^ fish are capable of producing *gnrh3* mRNA (though truncated) but not Gnrh3.

### The Effects of Lack of Gnrh3 on the HPG Axis

#### Developmental and Adult Expression Profiles of Reproductively Relevant Genes

**Fig 4:**
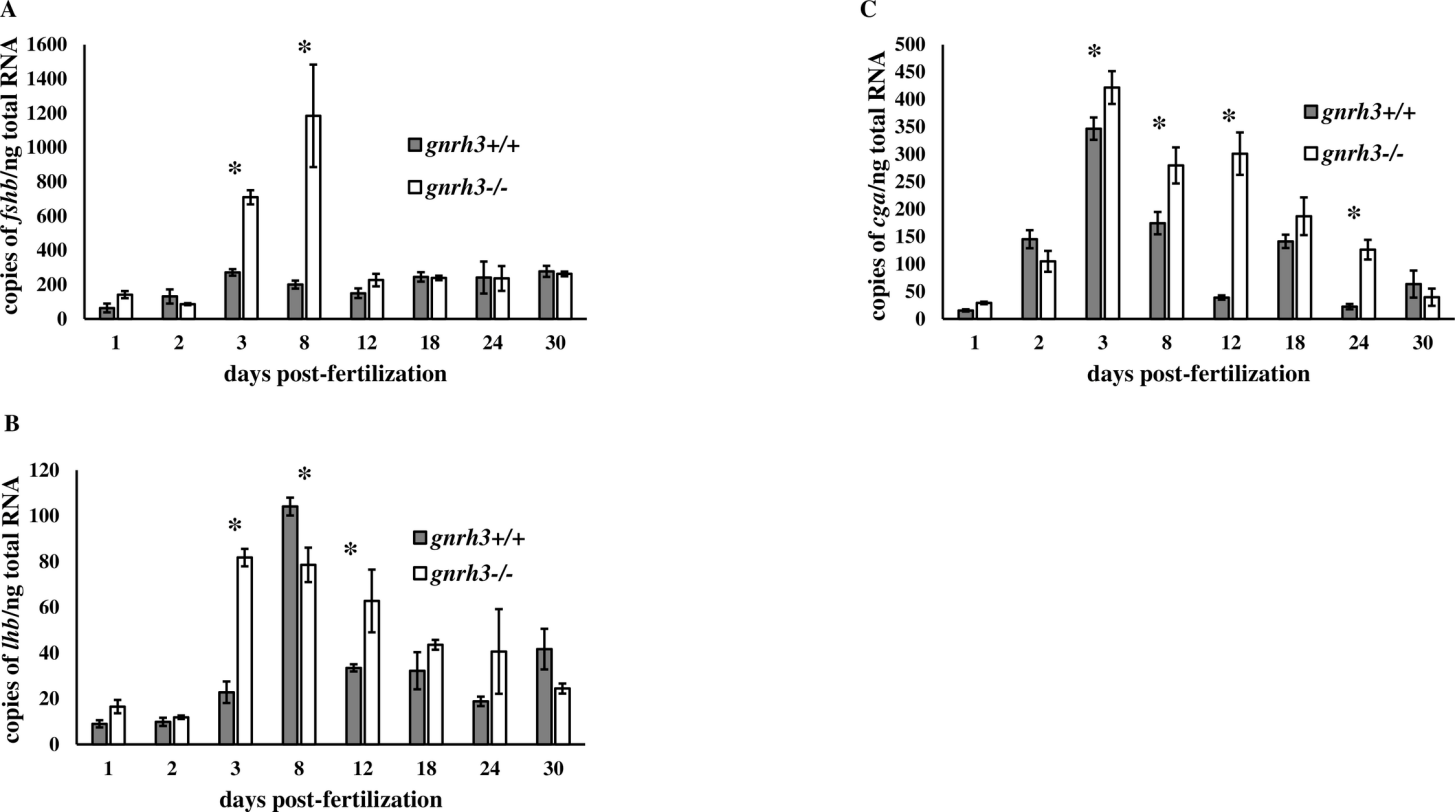
Developmental mRNA levels of pituitary gonadotropin genes tend to be higher in *gnrh3*^*-/-*^ fish than in *gnrh3*^*+/+*^ fish. mRNA levels at different time points of (A) *fshb*, (B) *lhb*, and (C) *cga* were measured in pooled samples of whole embryos/larvae/juveniles. Absolute mRNA levels were normalized to *ef1a* levels and are presented as mean ± SEM. Differences between genotypes at a specific time point were determined by a one-tailed, homoscedastic Student *t*-test and are considered statistically significant when *P ≤ 0.05. *gnrh3*^*+/+*^, grey bars. *gnrh3*^*-/-*^, white bars.

**Fig 5:**
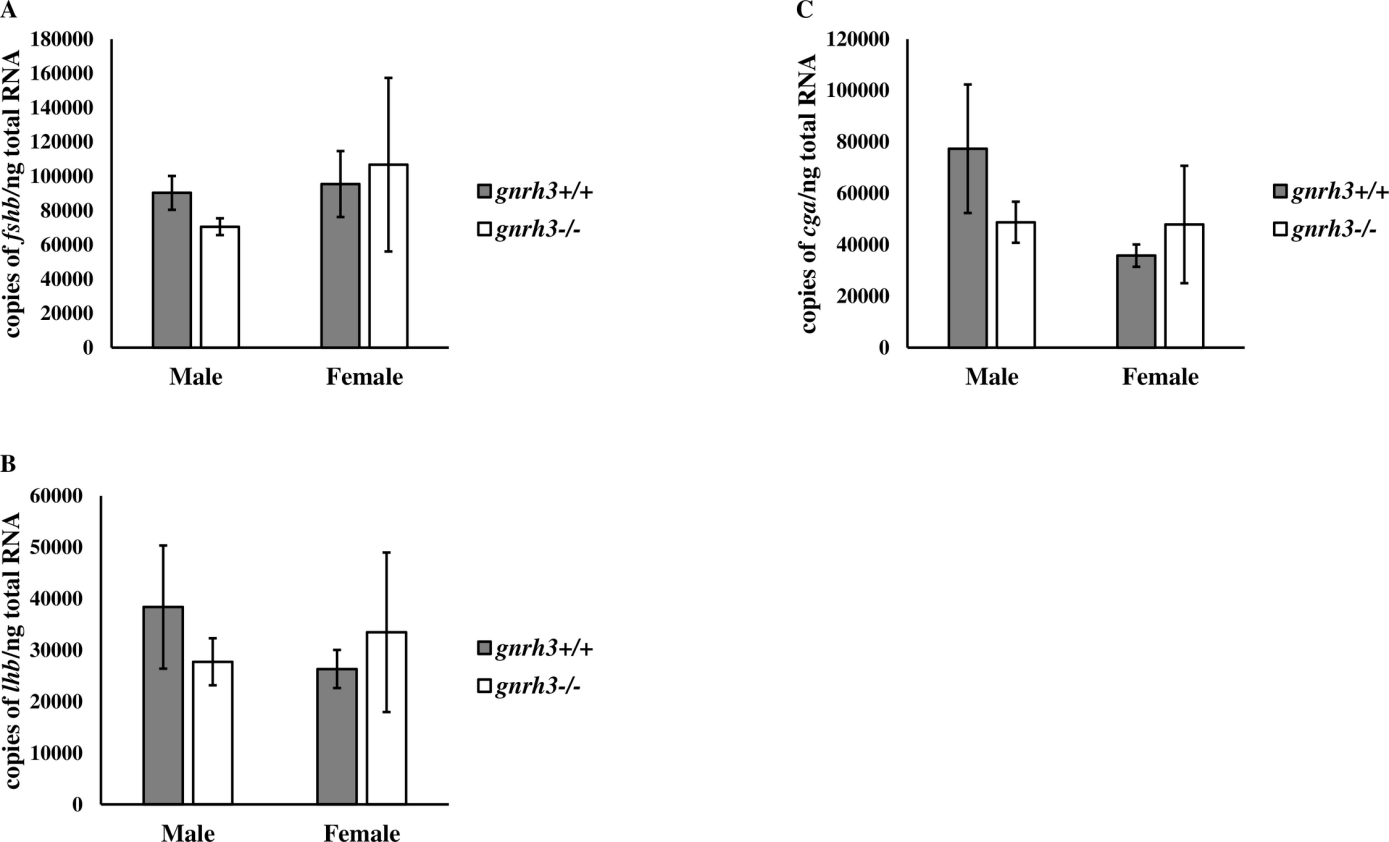
Male and female adult mRNA levels of pituitary gonadotropin genes show no differences between *gnrh3*^*+/+*^ and *gnrh3*^*-/-*^ fish. Pituitary mRNA levels of (A) *fshb*, (B) *lhb*, and (C) *cga* were determined using QPCR. Absolute mRNA levels were normalized to *ef1a* levels and are presented as mean ± SEM. Differences between genotypes for each sex were determined by a one-tailed, homoscedastic Student *t*-test and are considered statistically significant when *P ≤ 0.05. *gnrh3*^*+/+*^, grey bars. *gnrh3*^*-/-*^, white bars.

Throughout the first 30 days of development, we sampled *gnrh3*^*+/+*^ and *gnrh3*^*-/-*^ fish at eight time points to assess the mRNA levels of key genes involved in the reproductive axis. Three genes, in particular, that showed significant differences between *gnrh3*^*+/+*^ and *gnrh3*^*-/-*^ fish were the pituitary hormone genes *fshb* (Fig 4A), *lhb* (Fig 4B), and *cga* (Fig 4C). The two-way ANOVA revealed significant differences between genotype and sample time for all three genes. For the Student *t*-test for all three genes, *gnrh3*^*-/-*^ fish had higher expression levels at 1, 3, and 8 dpf, except for *lhb* at 8 dpf in which *gnrh3*^*+/+*^ mRNA levels were higher. At 12 dpf, both *lhb* and *cga* levels were also higher in the *gnrh3*^*-/-*^ fish, and at 24 dpf, this was also the case for *cga*. The mRNA levels of *fshb*, *lhb*, and *cga* in *gnrh3*^*-/-*^ fish were higher by 123–492%, 87–268%, and 21–692%, respectively, over their levels in *gnrh3*^*+/+*^ fish. Overall, *gnrh3*^*-/-*^ fish typically had higher mRNA levels of the three pituitary gonadotropin genes during development than *gnrh3*^*+/+*^ fish. When examined in adults, no significant differences in the expression of *fshb* (Fig 5A), *lhb* (Fig 5B), and *cga* (Fig 5C) in male and female *gnrh3*^*-/-*^ adults were obtained when compared with their *gnrh3*^*+/+*^ counterparts.

#### Pituitary Lh Content (determined by ELISA)

**Fig 6:**
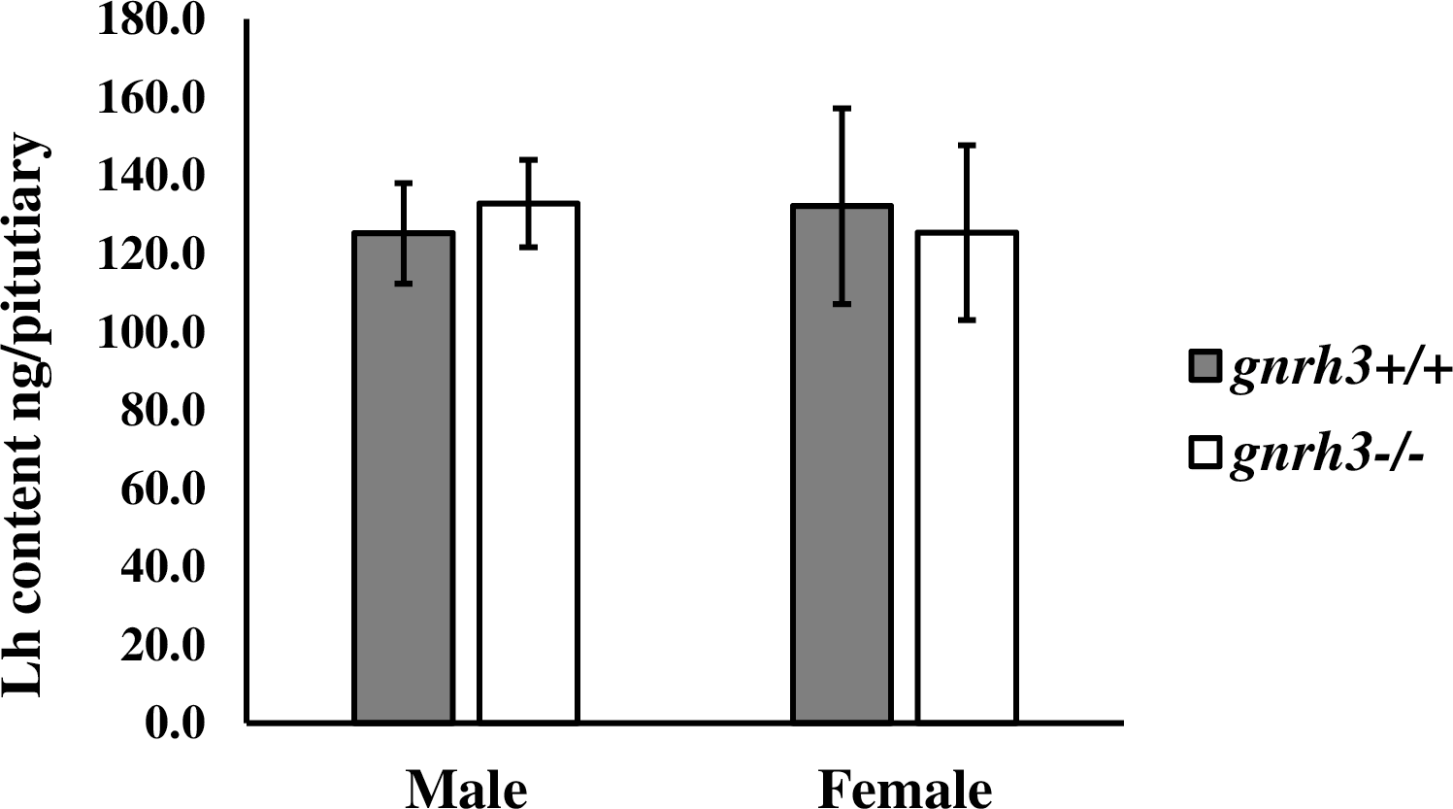
Adult male and female Lh levels in the pituitaries of *gnrh3*^*-/-*^ fish do not differ from *gnrh3*^*+/+*^ fish. Lh levels in the pituitaries of male and female adults (n = 4) were measured by ELISA. Values are presented as mean ± SEM. Differences between genotypes for each sex were determined by a one-tailed, homoscedastic Student *t*-test and are considered statistically significant when *P ≤ 0.05. *gnrh3*^*+/+*^, grey bars. *gnrh3*^*-/-*^, white bars.

Pituitary Lh levels in both sexes were similar between *gnrh3*^*+/+*^ and *gnrh3*^*-/-*^ fish (Fig 6). Therefore, in adults, both mRNA and peptide levels of Lh are not affected by the functional loss of the *gnrh3* gene.

#### Imaging of Gnrh3 fibers in *gnrh3-/- gnrh3*:*tdTomato* Juveniles

**Fig 7:**
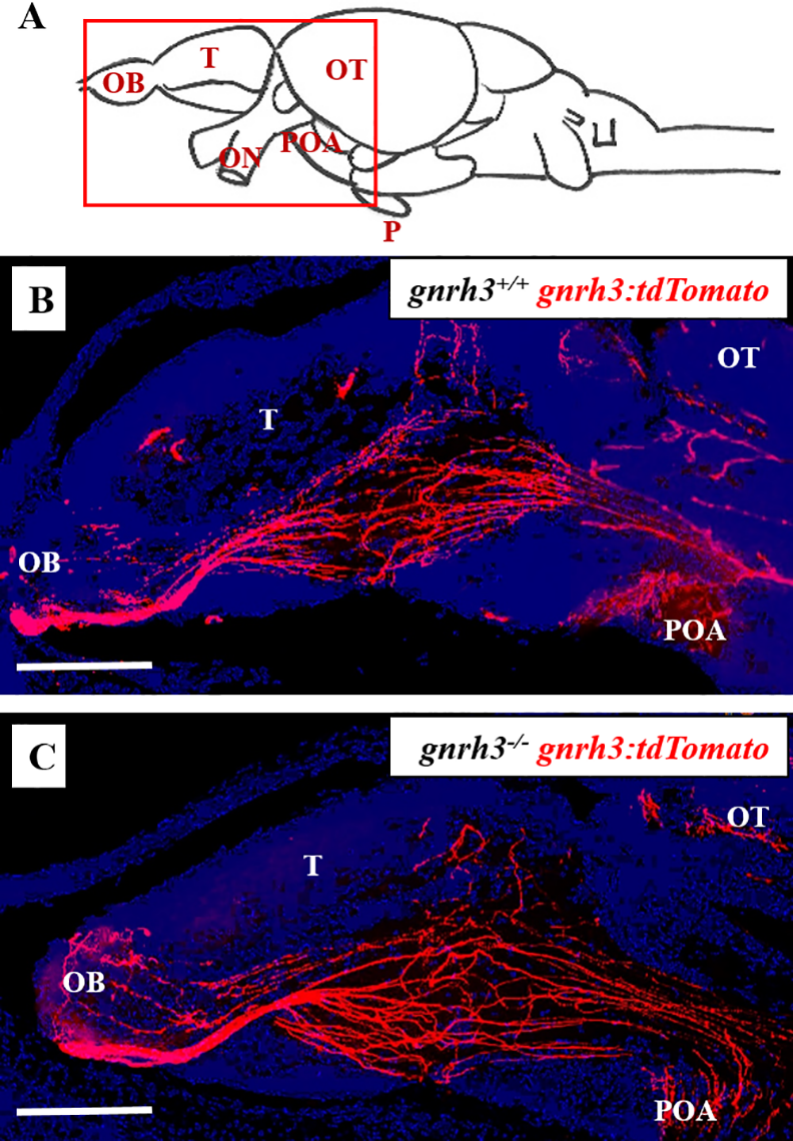
*gnrh3*^*-/-*^ juveniles exhibit normal Gnrh3 neuronal migration. In both *gnrh3*^*+/+*^ *gnrh3*:*tdTomato* (B) and *gnrh3*^*-/-*^ *gnrh3*:*tdTomato* (C) fish, Gnrh3-tdTomato-expressing soma located in the olfactory region and ventral telencephalon project fibers that extend posteriorly toward the hypothalamus and pituitary stalk (pituitaries not shown; A). Z-stack images were taken at 20x magnification on a Leica Microsystems DMi8 confocal microscope with a resolution of 1024 x 1024 and a z-step size of 0.10. All images were analyzed and assembled with Image J and Adobe Photoshop. Scale bars = 100 μm. OB = olfactory bulb. T = telencephalon. OT = optic tectum. ON = optic nerves. POA = pre-optic area. P = pituitary.

In order to determine whether *gnrh3*^*+/+*^ and *gnrh3*^*-/-*^ fish have different projecting patterns of Gnrh3 fibers during the development of the Gnrh3 system, we analyzed sagittal sections of *gnrh3*:*tdTomato* brains from both *gnrh3*^*+/+*^ and *gnrh3*^*-/-*^ juveniles. Both genotypes revealed Gnrh3-tdTomato-expressing soma in the olfactory bulbs and ventral telencephalon and fibers that extended from the soma to the hypothalamus and pituitary stalk (Fig 7B and 7C). The appearance of the *gnrh3*^*+/+*^ *gnrh3*:*tdTomato* transgenic signal was very similar to that reported previously [11,13]. Overall, there were no major differences observed between the *gnrh3*^*+/+*^ *gnrh3*:*tdTomato* and *gnrh3*^*-/-*^ *gnrh3*:*tdTomato* juvenile brain sections. These results indicate that Gnrh3 fibers (that would normally contain Gnrh3 peptide) in *gnrh3*^*-/-*^ fish do not undergo abnormal projecting patterns or, if they do, are at least corrected by 34 dpf.

### The Effects of Lack of Gnrh3 on Reproduction

#### Gonadal Histology

**Fig 8:**
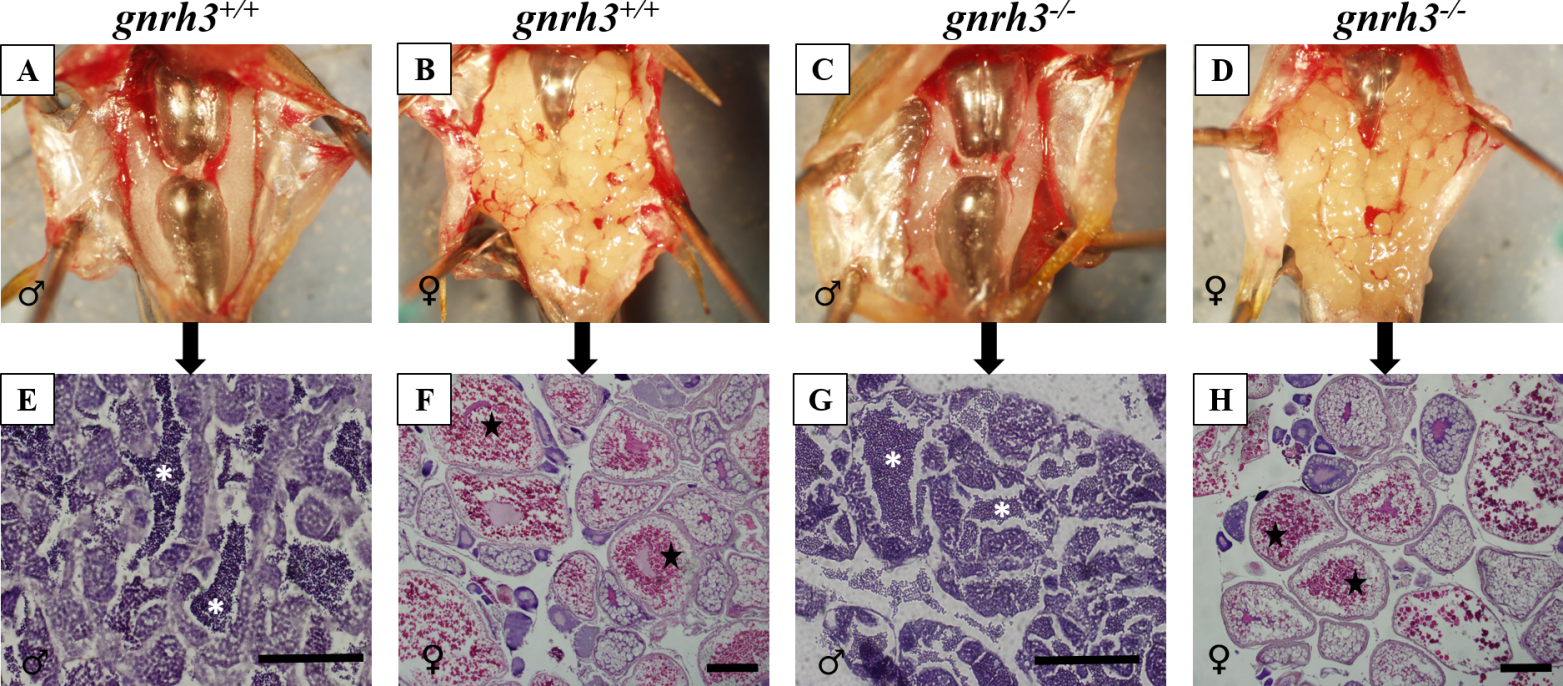
Adult gonadal morphology and gametogenesis do not differ between *gnrh3*^*+/+*^ and *gnrh3*^*-/-*^ fish for both males and females. (A-D) Gross gonadal morphology of adult male (A,C) and female (B,D) fish of both genotypes: *gnrh3*^*+/+*^ (A,B) and *gnrh3*^*-/-*^ (C,D). (E-H) Gonadal histology with hematoxylin and eosin staining, demonstrating no differences between *gnrh3*^*+/+*^ and *gnrh3*^*-/-*^ gametogenesis for both males and females. Male *gnrh3*^*-/-*^ testes contain spermatozoa in the lumens of testicular cysts (G), similar to *gnrh3*^*+/+*^ testes (E). Female *gnrh3*^*-/-*^ ovaries contain all stages of vitellogenesis (H), including late-vitellogenic oocytes, which is similar to *gnrh3*^*+/+*^ ovaries (F). White asterisks = mature spermatozoa in lumens of spermatocysts. Black stars = mature, late vitellogenic oocytes. Scale bars = 125 μm (testes) and 250 μm (ovaries).

In both males and females, gross morphology of the gonads did not appear different between *gnrh3*^*+/+*^ and *gnrh3*^*-/-*^ fish (Fig 8A vs 8C and Fig 8B vs 8D). In the histology conducted on males, all stages of advanced spermatogenesis were present in testes from both *gnrh3*^*+/+*^ (Fig 8E) and *gnrh3*^*-/-*^ (Fig 8G) fish, which included mature spermatozoa in the lumens of the spermatocysts. In females, all stages of oogenesis were observed in ovaries from both *gnrh3*^*+/+*^ (Fig 8F) and *gnrh3*^*-/-*^ (Fig 8H) fish, consistent with the asynchronous oocyte development of zebrafish ovaries. The ovaries from both *gnrh3*^*+/+*^ and *gnrh3*^*-/-*^ fish included late vitellogenic oocytes that were near to final maturation and ovulation (Fig 8F and 8H).

#### Fecundity, Fertility, and Offspring Survival

**Fig 9:**
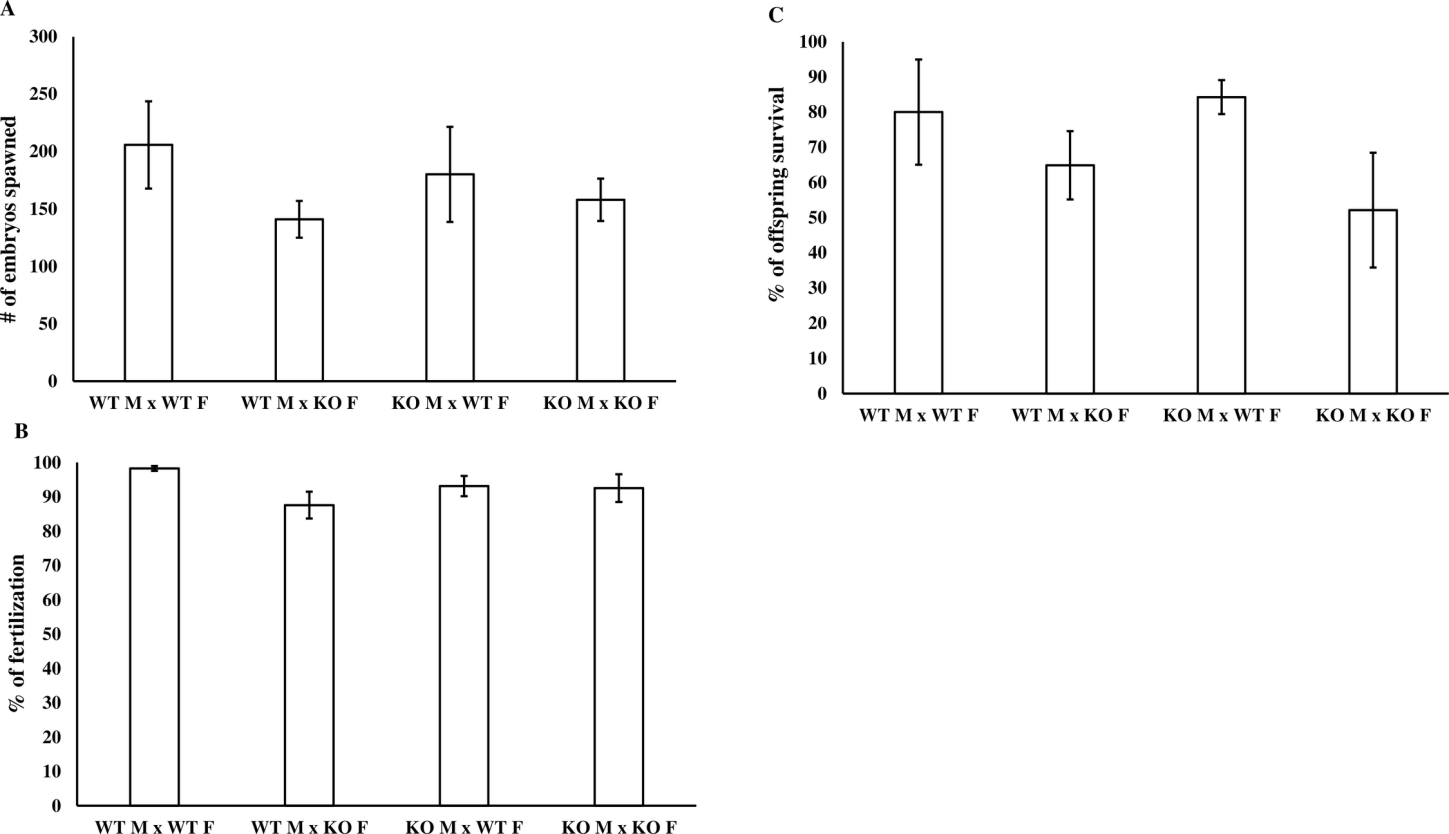
Reproductive capacity of *gnrh3*^*-/-*^ adult fish is not different from that of *gnrh3*^*+/+*^ fish. (A) Fecundity, (B) fertilization rate, and (C) offspring survival (to 2 dpf) were determined in the spawning of four different crossing combinations: *gnrh3*^*+/+*^ male x *gnrh3*^*+/+*^ female, *gnrh3*^*+/+*^ male x *gnrh3*^*-/-*^ female, *gnrh3*^*-/-*^ male x *gnrh3*^*+/+*^ female, and *gnrh3*^*-/-*^ male x *gnrh3*^*-/-*^ female. No differences between any of the parameters assessed were observed between any of the spawning combinations. All values are presented as mean ± SEM with 3–6 replicates per group, were subjected to a one-way ANOVA, and were considered statistically significant when *P ≤ 0.05. WT = *gnrh3*^*+/+*^. KO = *gnrh3*^*-/-*^. M = male. F = female.

Reproductive competence of *gnrh3*^+/+^ and *gnrh3*^*-/-*^ adult fish was assessed by measuring fecundity, fertilization rate, and offspring survival to 2 dpf. We in-crossed each genotype (male *gnrh3*^*+/+*^ x female *gnrh3*^*+/+*^ and male *gnrh3*^*-/-*^ x female *gnrh3*^*-/-*^) and out-crossed each genotype to the other genotype (male *gnrh3*^*-/-*^ x female *gnrh3*^*+/+*^ and male *gnrh3*^*+/+*^ x female *gnrh3*^*-/-*^). Each of the four spawning combinations included 3–6 spawning pairs. In observing the number of eggs produced by each spawning pair in one hour after the dividers were removed in the morning, we demonstrated no differences in fecundity between any of the crosses assessed (Fig 9A). Once the embryos were 4–5 hpf, we quantified the number of fertilized embryos to determine fertilization rates for each of the spawning combinations, which again did not differ between any of the four spawning combinations (Fig 9B). At 2 dpf, we counted the number of surviving embryos to determine offspring survival, and again, there were no differences between any of the four spawning combinations (Fig 9C). Therefore, the reproductive competence and capacity of *gnrh3*^*+/+*^ fish and *gnrh3*^*-/-*^ fish does not differ in terms of fecundity, fertility, and offspring survival.

## Discussion

The current study describes the generation and verification of a *gnrh3*^*-/-*^ zebrafish line and characterizes the components downstream of Gnrh3 in the reproductive HPG axis to reveal no major differences in the expression of key reproductive factors or in reproductive performance. We successfully developed a *gnrh3*^*-/-*^ line, using the TALEN technology, and verified this line at the levels of the gene, transcript, and protein. The mutant line harbored a large deletion in the *gnrh3* gene, which resulted in a lack of the Gnrh3 peptide in Gnrh3 neurons or elsewhere in the brain, while the incomplete mRNA was still detectable in these neurons. Developmental mRNA levels of pituitary gonadotropin genes in the *gnrh3*^*-/-*^ fish revealed higher levels for many time points; however, this effect was no longer visible by the time the fish reached adulthood. Surprisingly, the adult *gnrh3*^*-/-*^ fish had normal reproductive function, as evidenced by normal gametogenesis, spawning, and no changes in fecundity, fertility, and offspring survival. These findings strongly suggest that a compensatory mechanism is being activated in the *gnrh3*^*-/-*^ fish to mitigate the effects of the lack of Gnrh3, since Gnrh3 cell ablation causes infertility [12]. Therefore, we consider the possible explanations for the lack of the expected phenotype in the *gnrh3*^*-/-*^ fish and discuss potential compensatory factors.

The *gnrh3*^*-/-*^ line possesses a mutation in exon 2 of the *gnrh3* gene that results in a 62 bp discontinuous deletion that includes the start codon and the beginning of the nucleotide sequence encoding the Gnrh3 decapeptide. Because of the removal of the start codon and the lack of a methionine (in the same frame as Gnrh3) after the deletion and before the Gap, it appears that there is no correct translation of neither the Gnrh3 decapeptide nor the Gnrh3 Gap in the *gnrh3*^*-/-*^ fish. This is supported by the lack of protein signal in the *gnrh3*^*-/-*^ brain by both primary antibodies, anti-Gnrh3 Gap and anti-Gnrh3 decapeptide. Additionally, the lack of Gnrh3 neurons in the brain of *gnrh3*^*-/-*^ fish, including the preoptic area, is a strong indicator that there is no Gnrh3 in the pituitary. Therefore, there appears to be no Gnrh3 peptide in the *gnrh3*^*-/-*^ line.

The mutation found in the present study is drastically different than that found in the *hpg* mouse or in humans with hypogonadotropic hypogonadism due to *GNRH1* mutations. The *hpg* mouse possesses a 33.5 kb deletion that encompasses the 3’ regulatory elements and most of the GAP, while the decapeptide, the signal peptide, and the 5’ regulatory elements all remain intact [16]. Still, the decapeptide is not detected by immunostaining in GNRH1 cells [16]. Therefore, it is possible that the GAP is crucial for stabilizing the decapeptide in the *hpg* mouse [16]. Another possible explanation is that the missing 3’ region of the mouse *Gnrh* gene contains mRNA-stabilizing or translational elements that are required for successful translation of the entire gene [31]. In humans, however, the mutations found in individuals with hypogonadotropic hypogonadism tend to be smaller deletions (or substitutions) that cause frameshift mutations [17,18], which disrupt the translation of the functional product. The *gnrh3*^*-/-*^ zebrafish is the first organism with a targeted and heritable mutated *gnrh* gene that is not due to natural mutagenesis and also represents the first *gnrh* mutation of its size and location with the deletion of the start codon.

It is well-established that Gnrh is a major regulator of reproduction in many species of vertebrates [1,32–34], including zebrafish [12]. Gnrh3 is the hypophysiotropic form in zebrafish [9,11,35], and the elimination of Gnrh3-expressing cells during early zebrafish development (4–6 dpf) results in infertility [12]. Not only does the elimination of Gnrh3 negatively impact reproduction, it also impacts the ontogeny of Gnrh3 neurons. Transient *gnrh3* gene knockdown using anti-sense morpholino oligonucleotides resulted in misguided migration of Gnrh3 soma and fibers during neurogenesis [11]. The development and migration of the Gnrh3 neuron system is essential to its proper function as is seen in humans with Kallmann syndrome, in which a cessation of GNRH1 soma/fiber migration and subsequent hypogonadism are observed [36]. Based on these results, it was reasonable to assume that *gnrh3* knockout in zebrafish would have profound effects on the ontogeny of the Gnrh3 system and on reproductive performance. However, *gnrh3*^*-/-*^ fish revealed no misguided migration of Gnrh3 neurons, as what was observed in *gnrh3* knockdown [11]. Or, if misguided migration was present, it was corrected by at least 34 dpf. This finding and the results that *gnrh3*^*-/-*^ fish are reproductively normal demonstrate that the method used to remove functional gene products can have different effects on the resulting phenotypes.

Recently, much attention has been drawn to the fact that many gene knockout studies do not produce the expected phenotypes or those seen by knockdown studies using anti-sense morpholino oligonucleotides [37,38] or by cell ablation studies. Assuming that gene knockout activates a compensatory mechanism, while it is not activated by gene knockdown, the discrepancy may be caused by differences in the timing or the duration of the elimination that dictates whether compensation commences. More specifically, the transient presence of the antisense morpholino oligonucleotide in the system may be too short for the activation of the compensation, or the morpholino may be introduced too late and is not inherited, unlike what is observed in genetic knockouts. With regard to cell ablation, at least in the case of Gnrh3 soma [12], the treatment leading to phenotypes (given at 4–6 dpf) most likely misses the window of time in which the compensatory mechanism is primed (probably very early in ontogeny or upon Gnrh3 differentiation). Mayer and Boehm [39] illustrated the importance of the timing of treatment in a different system in mice. The kisspeptin/GPR54 system plays a major role in activating Gnrh neurons during murine puberty and reproduction [3,40–42]. The ablation of *Kiss1*- and *Gpr54*-expressing cells early in development results in fertile mice; therefore, the ablation was probably conducted early enough to allow a potential compensatory mechanism to be activated. The same treatment applied to adults resulted in infertility [39]. These results may suggest that a critical time window for the activation of potential compensation may exist in early development. It would be worthwhile to explore the possibilities of establishing conditional *gnrh3* gene silencing or Gnrh3 cell ablation in zebrafish to see how a later loss of functional Gnrh3 affects puberty and reproduction. Altogether, it is reasonable to conclude that a compensatory mechanism is being activated in the *gnrh3*^*-/-*^ fish at an early stage and that the loss of Gnrh3 primes this mechanism. Because cell ablation of Gnrh3 neurons yielded infertile fish [12], we propose that the compensatory mechanism that can activate the reproductive HPG axis most likely exists, at least partially, within Gnrh3 neurons and is being activated before the differentiation of Gnrh3 neurons.

Interestingly, the activation of a compensatory mechanism that ensures reproductive success in zebrafish is not limited to one gene and is seen in other reproductive neuropeptide genes. The best example is of kisspeptin/GPR54 signaling. While *Kiss1*^*-/-*^ mice [43] have been demonstrated to be infertile with hypogonadotropic hypogonadism, zebrafish *kiss1*^*-/-*^, *kiss2*^*-/-*^, and *kiss1*^*-/-*^ *kiss2*^*-/-*^ knockout lines exhibit no major differences in reproductive phenotypes [44]. Even the targeted mutations of *kiss1r* and *kiss2r* individually or together (*kiss1r*^*-/-*^ *kiss2r*^*-/-*^) do not disrupt gonadal development and reproductive performance in zebrafish [44], also differing from the results of *Gpr54*^*-/-*^ mice [45]. The inability of mice and humans to compensate for the loss of reproductive related factors in the central nervous system is puzzling, because at least mice are capable of activating compensatory mechanisms when other neuropeptides are mutated. For instance, serotonin and norepinephrine have been demonstrated to potentially compensate for a delayed growth phenotype in mice that do not produce functional neuropeptide Y [46]. Another example was seen by Li et al. [47], who found that phenotypes of embryonic fibroblasts from knockout mice are compensated by a functional homolog of the mutated gene. The combined results of the *gnrh3* and *kiss* genes may indicate that zebrafish activate a compensatory mechanism to ensure reproduction, whereas, although capable of for other genes, mice do not. It will be interesting to know if this also applies to other neuropeptides, such as neurokinin B—as mice and humans with mutated neurokinin B receptors are infertile [48,49]. The compensatory activation may be a common feature in lower vertebrates, as other teleost species display such compensation [50].

After finding significant changes in the developmental expression of pituitary gonadotropins in the *gnrh3*^*-/-*^ fish, we assessed the same genes in adulthood, studying males and females separately. For all genes in both sexes, there were no changes in mRNA levels between *gnrh3*^*+/+*^ and *gnrh3*^*-/-*^ fish. Consequently, some change occurred between early development and adulthood to allow the *gnrh3*^*-/-*^ pituitary gonadotropin mRNA levels to recover to WT levels. These results were also supported by the lack of changes in Gnrh3 fiber circuitry in juvenile *gnrh3*^*-/-*^ fish and the normal gametogenesis and reproduction in adult *gnrh3*^*-/-*^ fish. In general, gonadotropin mRNA levels are normally very low during development and gradually increase toward puberty to remain high throughout adulthood [51–55]. The differences observed between larval and adult *gnrh3*^*-/-*^ fish could be explained by an “adjustment period” for the compensatory factor, in which *gnrh3*^*-/-*^ levels are different during early development and are then adjusted to *gnrh3*^*+/+*^ levels, after the compensatory mechanism is fully primed, sometime between early development and adulthood.

Comparative analyses of gene silencing along multiple points of the HPG axis in zebrafish have revealed that silencing of genes upstream in the axis (*gnrh3*^*-/-*^ and *kiss1*^*-/-*^ *kiss2*^*-/-*^) [44] seems to have no major phenotypes compared to silencing of downstream genes (*fshb*^*-/-*^, *lhb*^*-/-*^, *fshr*^*-/-*^, and *lhr*^*-/-*^) [25,56,57], which have sex-specific effects but mostly result in delayed gametogenesis and sometimes infertility. It is possible then that compensatory mechanisms are more abundant at the level of the brain to overcome the loss of functional gene products than at the levels of the pituitary or gonads. This may be because many of the neuropeptides in the brain are pleiotropic, whereas the downstream elements are more functionally specialized. In addition, phenotypic differences between mice and zebrafish in silencing the hypophysiotropic *Gnrh1* [15] and *gnrh3*, respectively, and in silencing *Kiss1*/*Gpr54* [43,45] and *kiss*/*kissr* [44], respectively, may indicate that lower vertebrates are more capable of compensating for a lack of reproductively related neuropeptides within the HPG axis than more evolved vertebrates. This may be explained by the concept that many neuropeptides appear in multiple isoforms in lower vertebrates.

When considering other reproductively relevant factors that could compensate for a loss of Gnrh3, one of the most obvious candidates is the only other identified Gnrh isoform in zebrafish: Gnrh2. Gnrh2 is a midbrain tegmentum neuropeptide that is found in all vertebrates examined to date, except for rodents. The exact functions of Gnrh2 are not fully understood, but recent studies have implicated it in the regulation of feeding and reproductive behavior [58–59]. Based on the available information about Gnrh2, there are three possible scenarios in which Gnrh2 may contribute as a compensation factor in the absence of Gnrh3 in *gnrh3*^*-/-*^ fish: 1) Gnrh2 could be compensating from its soma in the midbrain tegmentum, since *gnrh2*:*eGFP* neurons project to the pituitary [13] and Gnrh2 peptide has been found in pooled zebrafish pituitaries, though at a much lower concentration than Gnrh3 [9]. In addition, Gnrh2 displayed a higher potency in activating the Gnrh receptor expressed in the pituitary than the hypophysiotropic Gnrh1 in the striped bass (*Morone saxatillis*) [60]. 2) Gnrh2 could be expressed in Gnrh3 neurons in the forebrain, because, as mentioned previously [12], some factor in the Gnrh3 soma is most likely regulating fertility (at least in females). 3) Alternatively, there could be a combination of these two scenarios that is contributing to the compensation in *gnrh3*^*-/-*^ fish. In order to determine if Gnrh2 solely compensates for reproductive performance when Gnrh3 is absent, we generated *gnrh3*^*-/-*^ *gnrh2*^*-/-*^ fish by crossing *gnrh3*^*-/-*^ fish with the *gnrh2*^*-/*-^ zebrafish line (unpublished results). Surprisingly, the double knockout fish were also fertile and did not display any major observable phenotypes in reproduction. Therefore, a factor that is not an identified Gnrh isoform is responsible for the compensation observed in our *gnrh3*^*-/-*^ *gnrh2*^*-/-*^ line.

The identity of the compensation factor and the mechanism by which it takes over the functions of Gnrh3 are still unknown. In addition to isoforms that often cross-activate each other’s receptors, other molecules that share some properties with the mutated factor could be responsible for the compensation. For instance, Rossi et al. [38] demonstrated the upregulation of a particular family of proteins (Emilin) in *egfl7* mutants, which both share a similar functional domain. Therefore, it is possible that the compensatory factor for Gnrh3 could be a peptide, possibly yet to be identified, that has structural similarities to the Gnrh3 decapeptide or to the Gnrh3 Gap. So far, known compensatory factors in the central nervous system include neurotransmitters, such as serotonin and norepinephrine, which compensate for a loss of functional neuropeptide Y in mice [46]. To this point, we have demonstrated that compensatory machinery still exists when Gnrh2 is eliminated in *gnrh3*^*-/-*^ fish. It will be interesting to discover the identity of the factor(s) utilized by the Gnrh3 compensatory system in zebrafish and whether the factor(s) compensating for Gnrh3 differ between early development, puberty, and adulthood.

In conclusion, the current study has provided the development and validation of a *gnrh3*^*-/-*^ line in zebrafish and characterized its endocrine components along the HPG axis and reproductive performance to reveal no major changes in reproductive phenotypes and competence. These results represent the first targeted and heritable mutation of a Gnrh isoform in any organism. The *gnrh3*^*-/-*^ zebrafish remains fertile, displaying normal reproductive performance, which is in stark contrast to phenotypes seen using other approaches (e.g., *gnrh3* knockdown, natural mutagenesis in mice and humans, ablation of Gnrh3-expressing cells) and/or other model systems (e.g., mice and humans). The results indicate that a compensatory mechanism is being activated, which is primed early on (probably upon Gnrh3 differentiation) and possibly within Gnrh3 neurons. In addition, the silencing of genes downstream in the zebrafish HPG axis have revealed more profound reproductive phenotypes, including impaired fertility. It is possible that lower vertebrates, like zebrafish, possess the ability to better compensate for a lack of important reproductive genes at the level of the brain but not necessarily at the levels of the pituitary and gonads. Potential compensation factors and sensitive windows of time for compensation during development, puberty, and adulthood should be further explored to determine how fish remain reproductively competent in the absence of a key hormone, such as Gnrh3.

## Supporting Information

S1 Fig Gnrh3 protein not detectable by another primary antibody in *gnrh3*^*-/-*^ adult brains. **(**A) Immunohistochemistry on adult coronal brain sections (Aa) using anti-zebrafish Gnrh3 decapeptide (red) demonstrates the presence of Gnrh3 signal in the *gnrh3*^*+/+*^ pre-optic area of the brain (Ab). However, no Gnrh3 signal was found in the *gnrh3*^*-/-*^ pre-optic area (Ac) or in any other region of the brain. Scale bars = 100 μm.
(TIF)
