# Supplementary figures and images for: Targeted Mutagenesis of the Hypophysiotropic Gnrh3 in Zebrafish (Danio rerio) Reveals No Effects on Reproductive Performance
Source: PLoS One. 2016 Jun 29;11(6):e0158141. doi: 10.1371/journal.pone.0158141 (PMC4927163; doi:10.1371/journal.pone.0158141)

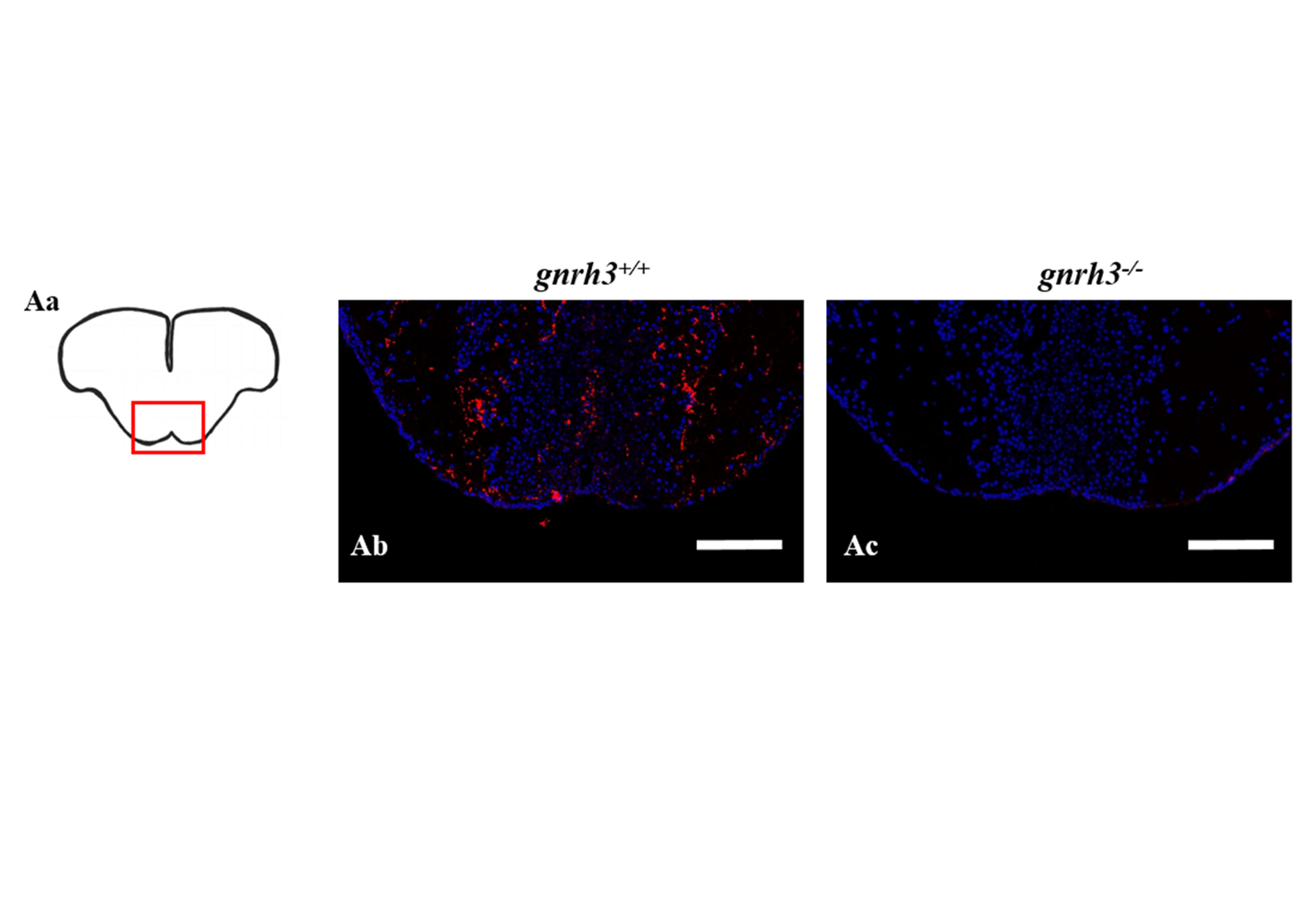

Supplement: S1 Fig — (A) Immunohistochemistry on adult coronal brain sections (Aa) using anti-zebrafish Gnrh3 decapeptide (red) demonstrates the presence of Gnrh3 signal in the gnrh3+/+ pre-optic area of the brain (Ab). However, no Gnrh3 signal was found in the gnrh3-/- pre-optic area (Ac) or in any other region of the brain. Scale bars = 100 μm. (TIF) [file pone.0158141.s001.tif]
